# Diagnosing Fanconi Anemia: A Rare Case Report From Rural India

**DOI:** 10.7759/cureus.63381

**Published:** 2024-06-28

**Authors:** Aashita Malik

**Affiliations:** 1 Pediatrics, Datta Meghe Institute of Higher Education and Research, Wardha, IND

**Keywords:** fanconi anemia, renal agenesis, mitomycin c, hypocellular marrow, bifid thumb

## Abstract

Fanconi anemia is a rare but most prevalent form of inherited aplastic anemia, predominantly transmitted in an autosomal recessive manner, except for one X-linked variant. It arises from mutations in the genes across 16 different complementation groups that are crucial for DNA stability. It is marked by a wide range of congenital malformations, progressive pancytopenia, and an increased risk of both hematological malignancies and solid tumors. The congenital abnormalities associated with it can affect various organ systems, including the skeletal system, with significant variability among patients. One similar case has been reported here, which had the typical clinical features of FA. Due to varied phenotypic presentation, diagnosing FA can be challenging. A Chromosomal Breakage Study using mitomycin C (MMC) or diepoxybutane (DEB) is a distinctive cellular marker that aids in the diagnosis.

## Introduction

First described in 1927 by Swiss pediatrician Guido Fanconi, Fanconi anemia (FA) is a rare, genetically inherited disorder primarily transmitted in an autosomal recessive manner. It is characterized by congenital malformations, progressive pancytopenia, cellular hypersensitivity to DNA cross-linking agents, and an increased risk of acute myelogenous leukemia (AML) and other malignancies [1]. Molecular diagnosis of FA is quite complex, not only because at least 15 genes are associated with its development but also because the mutation spectra of most FA-associated genes are very diverse and some of these genes frequently contain large deletions or duplications [2-4]. Developmental and physical abnormalities in FA encompass hyperpigmentation, short stature, thumb and forearm malformations, skeletal anomalies, small head or eyes, renal issues, hearing defects, heart disease, gastrointestinal problems, and hypogonadism [5,6].

## Case presentation

**Figure 1:**
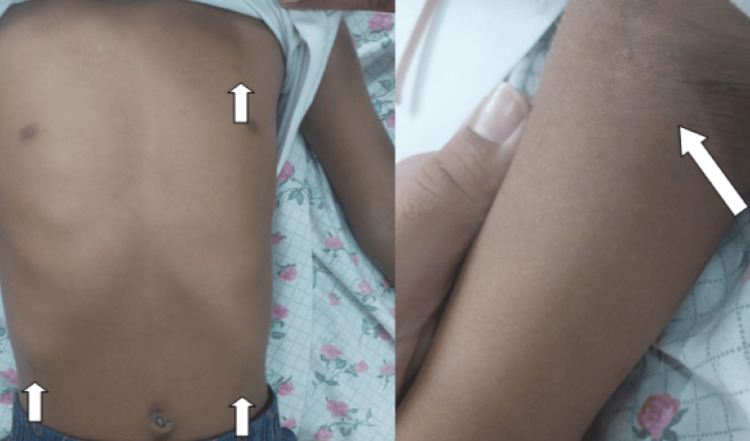
Depicting hypopigmented spots on the trunk and axilla with axillary freckling

A six-year-old female child, first by birth order, born out of a nonconsanguineous marriage, presented to the emergency department of our hospital with complaints of excessive fatiguability for the past two months. The father also noticed a gradual increase in the pallor on the child’s face. She was not eating well and had reduced activity. They consulted with some local doctors but no relief was attained. On admission, the child looked pale but comfortable. Her heart rate was 146 beats per minute, with pulses well felt and a normal respiratory rate and oxygen saturation. She was developmentally normal, with all milestones attained according to age. Anthropometry revealed a height of 110 cm and a weight of 14 kg. According to the Indian Academy of Pediatrics (IAP) weight for age, the child had grade 1 malnutrition. The head circumference was 46 cm, which was microcephaly for this age and sex. General examination revealed generalized hypopigmentation, with hypopigmented macules over the chest, axilla, and back, all measuring 0.2 x 0.3 x 0.2 cm each, most likely to be café au lait spots (Figure 1).

**Figure 2:**
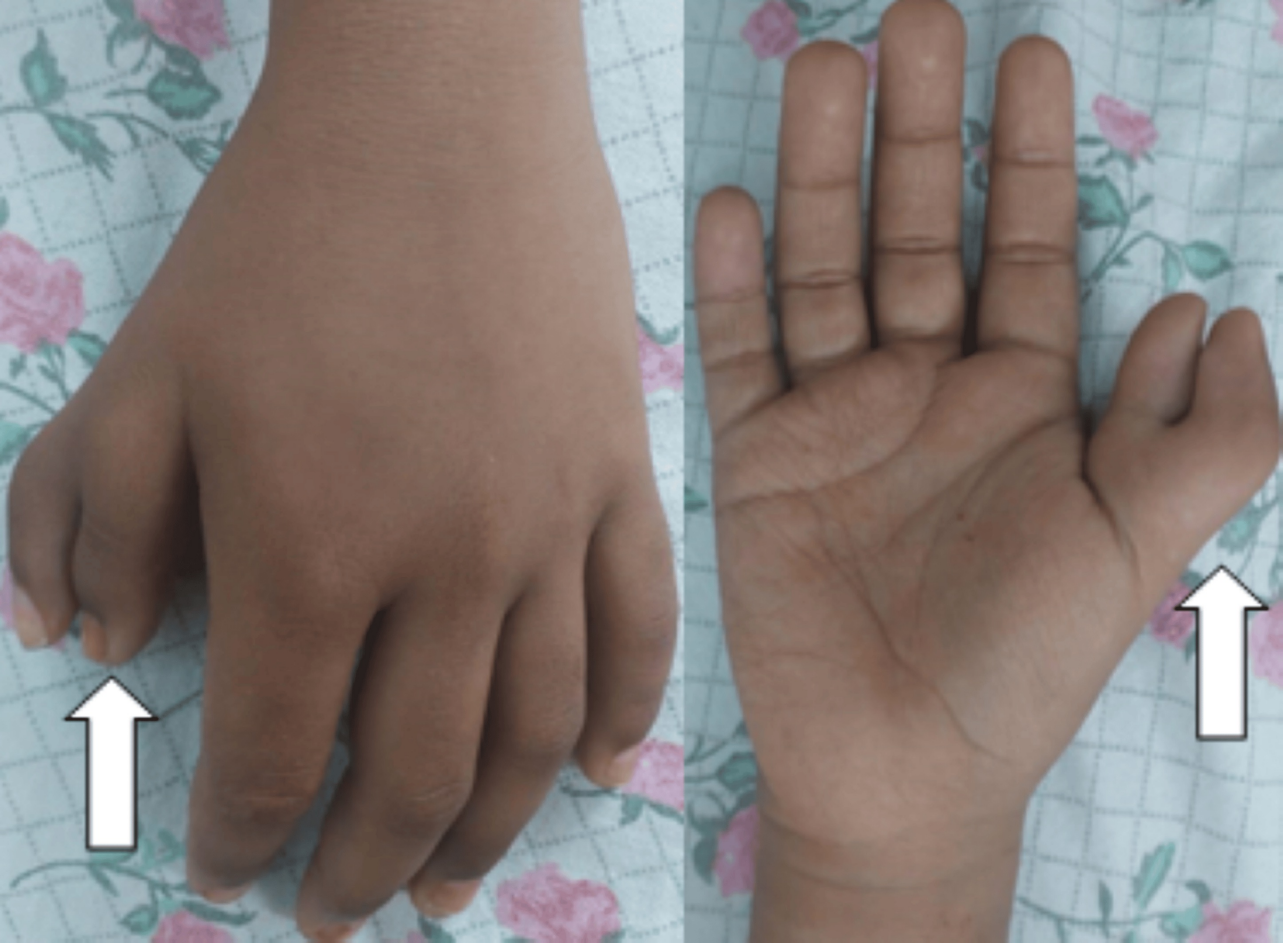
Right-sided bifid thumb

The patient had left-sided hypoplastic thumb and right-sided bifid thumb (Figure 2). 

The abdomen was soft, without any organomegaly. The rest of the systemic examination was within normal limits. Laboratory investigations were done, which revealed pancytopenia, along with normal hemolytic workup including liver function test, reticulocyte count, and direct Coombs test (Table 1).

**Table 1:** Laboratory investigations Hb: Hemoglobin; TLC: total leukocyte count

Investigation	Patient’s result	Biological reference value
Hb	6.9	13-15 g/dl
TLC	3600	4000-11000/cumm
Platelet count	38,000	150,000-450,000/cumm
Alkaline phosphatase	48	38-126 unit/l
Alanine transaminase	25	<50 U/l
Aspartate transaminase	22	17-59 U/l
Total protein	7.2	6.3-8.2 g/dl
Albumin	4	3.5-5 g/dl
Total bilirubin	0.3	0.2-1.3 mg/dl
Unconjugated bilirubin	0.2	0-0.3 mg/dl
Conjugated bilirubin	0.1	0-1.1 mg/dl
Globulin	3.2	2.3-3.5 mg/dl
Reticulocyte count	1.8	0.5-2.5 %
Urea	15	9-20 mg/dl
Creatinine	0.6	0.6-1.2 mg/dl
Sodium	142	137-145 mmol/l
Potassium	4.1	3.5-5.1 mmol/l
Direct Coombs test	Negative	-

Based on the clinical impression, the differential diagnosis of FA, inherited bone marrow failure, and acquired aplastic anemia were considered. Ultrasound abdomen-pelvis was done suggestive of empty left renal fossa, most likely due to left renal agenesis. The right kidney was seen at normal anatomical location and was of the appropriate size. High-performance liquid chromatography (HPLC) was sent, and bone marrow examination was done. As the reports were awaited, the child was transfused with one unit of packed red blood cell (PRBC) and two units of platelets. A repeat complete blood count (CBC) was done suggestive of hemoglobin (Hb) = 10 g/dl, total leukocyte count (TLC) = 1800 cumm, and platelet count = 50,000 cumm. In view of low platelets, the patient was started on Tab eltrombopag 50 mg and Inj filgrastim was started for decreased TLC. The HPLC report was suggestive of HbF = 9.5%, HbA0 = 88.6%, HbA2 = 1.9% which was interpreted as either hereditary persistence of fetal hemoglobin (HPFH) trait or delta beta thalassemia trait. The bone marrow biopsy showed hypocellular marrow with depletion of all cell lines suggestive of aplastic anemia. Suspecting FA, chromosomal breakage test was done which revealed high chromosomal breakage in mitomycin C (MMC)-induced peripheral blood culture (Table 2).

**Table 2:** Chromosomal breakage study revealed high chromosomal breakage in mitomycin C-induced peripheral blood culture as compared to controls

	Spontaneous	Mitomycin C-induced
Patient	50 cells = 0.00/cell; cells with radials = none	50 cells = 10.5 brks/cell; cells with radials = 4
Control	50 cells = 0.00/cell; cells with radials = none	50 cells = 0.048 brks/cell ; cells with radials = none

The parents were explained about the condition and the need for bone marrow transplantation as a permanent cure. Human leukocyte antigen (HLA) typing of sibling and parents was advised, but the parents were not willing for the same.

## Discussion

FA is a rare genetic disorder characterized by a range of abnormalities, including absent limbs, absent kidneys, anemia, urogenital anomalies, and bone marrow aplasia. It results from mutations or defects in specific genes. The protein produced by these genes is crucial for recognizing and repairing damaged DNA. In FA, due to these genetic defects, the DNA repair process is impaired, preventing the production of new stem cells and leading to aplastic anemia [7]. In a case by Sharma et al. diagnosis was based on physical abnormalities, blood investigations, and bone marrow examination along with the use of a chromosomal breakage test for confirmation [8]. In a study by Alahmadi et al., the mean age of presentation of FA was seven to eight years with a male preponderance [9]. In our case, however, the patient is a female. A study by Sathyanarayana et al. showed that FA is associated with renal anomalies [10]. In the current case also, there was left renal agenesis. The complications of FA are bone marrow failure, acute myeloid leukemia, myelodysplastic syndromes, and solid tumors of the head and neck, skin, gastrointestinal tract, and genital tract. FA patients require transfusion of RBCs at frequent intervals [11-13]. Additional therapies for FA include the use of androgens to increase Hb and platelet counts, and granulocyte colony-stimulating factor (G-CSF) or granulocyte-macrophage colony-stimulating factor (GM-CSF) to treat neutropenia. The definitive treatment for marrow failure in FA patients is a bone marrow transplant. Preventive strategies involve prenatal testing and family planning. Prenatal testing methods include fetal ultrasonography, molecular genetic testing through amniocentesis or chorionic villus sampling, and chromosomal breakage studies using diepoxybutane (DEB) or MMC. Family planning measures consist of genetic counseling for young adults who are affected, carriers, or at risk of being carriers.

## Conclusions

FA in pediatric patients presents a complex and challenging condition characterized by a broad spectrum of congenital abnormalities, progressive pancytopenia, and an increased risk of malignancies. Early diagnosis through chromosomal breakage studies and genetic testing is crucial for effective management and improved outcomes. Treatment strategies, including androgens, G-CSF, GM-CSF, and ultimately bone marrow transplantation, can significantly enhance the quality of life and prognosis for affected children. Prenatal testing and genetic counseling are essential for at-risk families to manage and mitigate the impact of FA. This case underscores the importance of a multidisciplinary approach in the diagnosis, treatment, and prevention of FA in pediatric patients.
